# Chronotropic incompetence is associated with reduced aerobic conditioning and sedentary behavior in patients with post-acute COVID-19 syndrome

**DOI:** 10.1590/S1678-9946202466032

**Published:** 2024-05-13

**Authors:** Milena dos Santos Barros Campos, Gabriela Menezes Gonçalves de Brito, Karinne Simões da Cruz Santos, Marcos Antonio Almeida Santos, Paulo Ricardo Martins-Filho, Antônio Carlos Sobral Sousa

**Affiliations:** 1 Universidade Federal de Sergipe, Programa de Pós-Graduação em Ciências da Saúde, Aracaju, Sergipe, Brazil; 2 Universidade Tiradentes, Departamento de Medicina, Aracaju, Sergipe, Brazil; 3 Universidade Federal de Sergipe, Hospital Universitário, Divisão de Cardiologia, Aracaju, Sergipe, Brazil; 4 Rede D’Or São Luiz, Clínica e Hospital São Lucas, Aracaju, Sergipe, Brazil; 5 Universidade Tiradentes, Programa de Pós-Graduação em Saúde e Meio Ambiente, Aracaju, Sergipe, Brazil; 6 Universidade Federal de Sergipe, Laboratório de Patologia Investigativa, Aracaju, Sergipe, Brazil

**Keywords:** COVID-19, Post-acute COVID-19 syndrome, Long COVID-19, Exercise test, Cardiovascular system, Sedentary behavior

## Abstract

Post-acute COVID-19 syndrome, or long COVID, presents with persistent symptoms, including cough, dyspnea, and fatigue, extending beyond one month after SARS-CoV-2 infection. Cardiac complications such as chest pain and arrhythmias have raised concerns, with chronotropic incompetence (CI), an inadequate heart rate increase during exercise, emerging as a significant condition contributing to diminished exercise tolerance and quality of life. This study estimated the prevalence of CI and explored its association with aerobic capacity and physical activity levels in long COVID patients. A cross-sectional study was conducted at a private hospital in Sergipe, Brazil, involving 93 patients over 18 years old with persistent post-COVID-19 symptoms after confirmed SARS-CoV-2 infections. Exclusion criteria included beta-blocker use, inadequate respiratory exchange ratio, and inability to complete cardiopulmonary exercise testing (CPET). Clinical histories, CPET results, and chronotropic index calculation were used to identify CI, with logistic regression analyzing associated factors. Of the participants (mean age 45 years; average duration since COVID-19 diagnosis 120 days), 20.4% were diagnosed with CI. Logistic regression identified a strong association between CI and sedentary behavior (OR 11.80; 95% CI 2.54 to 54.78; p=0.001). Patients with CI showed lower predicted peak heart rates and maximal oxygen uptake. The prevalence of CI among long COVID patients in this study was approximately 20%, associated with decreased aerobic capacity and increased sedentary behavior. These findings highlight the need for timely diagnosis and therapeutic interventions, including cardiopulmonary rehabilitation, to enhance the quality of life in post-COVID patients with CI. The study’s cross-sectional design and its specific context have limited causality inference and generalizability, underscoring the importance of further research in diverse settings.

Post-acute COVID-19 syndrome, commonly referred to as long COVID, is characterized by persistent symptoms such as cough, dyspnea, and fatigue beyond one month following SARS-CoV-2 infection^1^. Alongside with these symptoms, there is a growing concern about the cardiac complications experienced by these patients, with recent evidence showing a high prevalence of chest pain and arrhythmia^2^. Additionally, studies have not only found a significant decline in the aerobic condition of these individuals^3^ but also identified an increased risk for chronotropic incompetence, a condition defined as an insufficient increase in heart rate to meet metabolic demands^4^. Such cardiac manifestations can lead to exercise intolerance, a deterioration in quality of life, and an increased risk of cardiovascular events and mortality, underscoring the need for a deeper understanding of cardiac involvement in long COVID. Here, we estimated the prevalence of chronotropic incompetence and its association with aerobic capacity and physical activity in long COVID patients.

Our cross-sectional study, conducted at a private hospital in Sergipe State, Brazil, involved patients aged 18 and older with persistent post-COVID-19 symptoms and a prior confirmed SARS-CoV-2 diagnosis via RT-PCR. We excluded patients taking beta-blockers, those with a respiratory exchange ratio (RER) of less than 1.0 during maximal exercise, and individuals unable to complete cardiopulmonary exercise testing (CPET). Anticipating a 20% prevalence of chronotropic incompetence in post-COVID-19 syndrome patients^5^, and with 120 eligible patients at the hospital, we determined a minimum sample size of 81, factoring in a 1.96 critical value and a 5% sample error.

Participants underwent a detailed clinical history assessment, including data on sex, age, body mass index, sedentary behavior, comorbidities, medication use, infection severity, and time since COVID-19 diagnosis. Sedentary individuals were defined as those engaging in less than 150-300 minutes of moderate-intensity or 75-150 min of vigorous-intensity aerobic physical activity per week, or an equivalent combination^6^. After the clinical history evaluation, patients underwent a resting electrocardiogram and CPET. The cardiopulmonary exercise test results included systolic and diastolic blood pressure, heart rate, oxygen saturation, maximal oxygen uptake, oxygen pulse, oxygen uptake at the anaerobic threshold, oxygen consumption recovery, the regression slope relating minute ventilation to carbon dioxide output, RER, maximal voluntary ventilation, oxygen uptake efficiency slope, and cardiorespiratory optimal point. Test termination criteria followed the guidelines set by the III Brazilian Cardiology Society’s Ergometric Tests Guidelines.

Our outcome of interest was the chronotropic index, calculated as (HR_peak_ - HR_rest_) / (Predicted HR_peak_ - HR_rest_). Patients with a score of 0.8 or lower were deemed to have chronotropic incompetence^7^. We determined the prevalence of chronotropic incompetence by dividing the number of patients with this condition by the total number assessed, calculating its 95% confidence interval using the Wilson method. To compare CPET results between patients with and without chronotropic incompetence, we used the Mann-Whitney test, setting a significance level of 5%. Furthermore, a logistic regression model with backward selection was applied to identify factors associated with chronotropic incompetence among post-COVID-19 syndrome patients, with a significance level set at 5%. To assess the strength and direction of the association between variables and chronotropic incompetence, we used the odds ratio (OR) with a 95% confidence interval (95% CI). Analyses were conducted using JASP software (version 0.13, JASP Team, Amsterdam, Netherlands).

Initially, 120 patients with post-COVID-19 syndrome were assessed for eligibility. Exclusions included 14 on beta-blockers and 13 with an RER below 1.0, leading to 93 volunteers who completed the CPET protocol and were included in the analysis. The participants’ mean age was 45 years, and the average duration since the COVID-19 diagnosis was 120 days (ranging from 90 to 240 days). Among them, 19 individuals (20.4%, 95% CI 13.5% to 29.7%) were diagnosed with chronotropic incompetence. These patients exhibited lower predicted peak heart rates and maximal oxygen uptake (Table 1). Logistic regression analysis revealed a strong association between chronotropic incompetence and sedentary behavior (OR 11.80, 95% CI 2.54 to 54.78; p = 0.001) (Table 2).

**Table 1:** Cardiopulmonary exercise test results among post-COVID-19 syndrome patients with and without chronotropic incompetence.

Variables	Chronotropic incompetence		p-value

	Yes (n=19)	No (n=74)	
**Baseline measurements**			
Systolic blood pressure at rest (SBPrest) (mmHg)	130.0 (120.0 – 140.0)	130.0 (120.0 – 140.0)	0.589
Diastolic blood pressure at rest (DBPrest) (mmHg)	80.0 (80.0 – 90.0)	80.0 (80.0 – 90.0)	0.256
Heart rate at rest (HRrest) (beats/minute)	79.0 (65.5 – 86.5)	75.0 (68.0 – 85.0)	0.561
Peripheral measured oxygen saturation at rest (SPO_2_rest) (%)	98.0 (97.0 – 98.5)	97.0 (97.0 – 98.8)	0.470
Forced expiratory volume at 1 second (FEV1) (%)	80.4 (75.2 – 93.3)	89.0 (82.7 – 94.0)	0.070
Forced vital capacity (FVC) (%)	89.0 (81.5 – 95.0)	92.5 (87.1 – 100.0)	0.126
Tiffeneau-Pinelli index (FEV1/FVC ratio)	75.0 (73.2 – 84.0)	78.0 (75.9 – 81.8)	0.196
Forced expiratory flow (FEF25-75%) (%)	89.8 (74.9 – 101.5)	96.0 (76.2 – 106.8)	0.386
**Cardiopulmonary exercise test results**			
Peak systolic blood pressure (SBPpeak) (mmHg)	190.0 (170.0 – 200.0)	190.0 (190.0 – 200.0)	0.296
Peak diastolic blood pressure (DBPpeak) (mmHg)	90.0 (80.0 – 100.0)	90.0 (80.0 – 100.0)	0.352
Predicted peak heart rate (predicted HRpeak) (%)	86.0 (84.0 – 87.5)	98.5 (95.0 – 103.8)	< 0.001*
Heart rate recovery at 1 minute (HRR1) (beats/minute)	24.0 (17.0 – 26.0)	25.0 (21.3 – 30.0)	0.138
Peripheral measured oxygen saturation at peak exercise (SPO_2_peak) (%)	97.0 (94.0 – 98.0)	96.0 (95.0 – 97.0)	0.500
Maximal oxygen uptake (V^•^O_2_max) (ml.Kg^-1^. min^-1^)	20.4 (17.6 – 26.4)	26.1 (21.8 – 34.9)	0.006*
Predicted maximal oxygen uptake (predicted V^•^O_2_max) (%)	69.0 (62.5 – 80.0)	79.0 (69.3- 91.8)	0.011*
Oxygen pulse (O_2_pulse) (mL/beat)	12.6 (9.9 – 15.3)	13.3 (9.8 – 15.6)	0.939
Peak percentage of predicted oxygen pulse (predicted O_2_-PulsePeak) (%)	81.0 (74.5 – 96.5)	84.5 (72.5 – 92.0)	0.939
Oxygen uptake at the anaerobic threshold (AnT) (%)	60.0 (56.5 – 69.0)	59.0 (51.3 – 66.0)	0.692
Oxygen consumption recovery (V^•^O_2_rec) (seconds)	90.0 (90.0 – 108.8)	100.0 (90.0 – 100.0)	0.377
Regression slope relating minute ventilation to carbon dioxide output (V^•^E/V^•^CO_2_ slope)	32.9 (30.7 – 34.9)	33.2 (31.0 – 3.9)	0.801
Respiratory exchange ratio (RER)	1.08 (1.05 – 1.13)	1.12 (1.05 – 0.13)	0.123
Maximal voluntary ventilation (MVV) (liters/minute)	82.4 (60.4 – 96.5)	92.2 (67.7 – 109.1)	0.176
Predicted maximal voluntary ventilation (predicted MVV) (%)	75.0 (64.0 – 83.5)	77.5 (72.1 – 84.0)	0.303
Oxygen uptake efficiency slope (OUES) (liters/minute)	1.9 (1.6 – 2.5)	2.2 (1.7 – 3.0)	0.185
Cardiorespiratory optimal point (COP)	22.7 (20.1 – 25.5)	22.4 (20.6 – 24.5)	0.685

*p-values less than 0.05 were considered statistically significant.

**Table 2:** Univariate and multivariate analyses of factors associated with chronotropic incompetence among patients with post-COVID-19 syndrome.

Variables	n	Chronotropic incompetence		Univariate analysis		Multivariate analysis (final model)	

		Yes (n=19)	No (n=74)	OR (95% CI)	p-value	OR (95% CI)	p-value
**Sex**							
Male	61	12 (19.7)	49 (80.3)				
Female	32	7 (21.9)	25 (78.1)	0.88 (0.31 – 2.50)	0.802		
**Age**							
≤ 45 years	47	12 (25.5)	35 (74.5)				
> 45 years	46	7 (15.2)	39 (84.8)	0.53 (0.19 – 1.48)	0.222	0.41 (0.13 – 1.28)	0.125
**Body mass index**							
≤ 27 kg/m^2^	44	7 (15.9)	37 (84.1)				
> 27 kg/m^2^	49	12 (24.5)	37 (75.5)	1.71 (0.61 – 4.84)	0.309		
**Sedentarism**							
No	45	2 (4.44)	43 (95.6)				
Yes	48	17 (35.4)	31 (64.6)	11.80 (2.54 – 54.78)	0.002	13.1 (2.76 – 62.28)	0.001*
**Diabetes mellitus**							
No	86	18 (20.9)	68 (79.1)				
Yes	7	1 (14.3)	6 (85.7)	0.63 (0.07 – 5.57)	0.677		
**Hypertension**							
No	59	13 (22.0)	46 (78.0)				
Yes	34	6 (17.6)	28 (82.4)	0.76 (0.26 – 2.22)	0.614		
**Arrhythmia**							
No	52	13 (25.0)	39 (75.0)				
Yes	41	6 (14.6)	35 (85.4)	0.51 (0.18 – 1.50)	0.223		
**Angiotensin receptor blockers**							
No	67	14 (20.9)	53 (79.1)				
Yes	26	5 (19.2)	21 (80.8)	0.90 (0.29 – 2.82)	0.858		
**Calcium channel blockers**							
No	82	17 (20.7)	65 (79.3)				
Yes	11	2 (18.2)	9 (81.8)	0.85 (0.17 – 4.30)	0.844		
**Use of corticosteroids**							
No	86	18 (20.9)	68 (79.1)				
Yes	7	1 (14.3)	6 (85.7)	0.63 (0.07 – 5.57)	0.677		
**COVID-19 severity**							
Mild-moderate	71	13 (18.3)	58 (81.7)				
Severe	22	6 (27.3)	16 (72.7)	1.67 (0.55 – 5.10)	0.365		
**Time since COVID-19 diagnosis**							
≤ 3 months	59	11 (18.6)	48 (81.4)				
> 3 months	34	8 (23.5)	26 (76.5)	1.34 (0.48 – 3.75)	0.574		

OR = odds ratio; CI = confidence interval; *p-values less than 0.05 were considered statistically significant.

The findings of our investigation are consistent with those from a cohort study based in San Francisco that assessed cardiopulmonary symptoms in patients one year after SARS-CoV-2 infection. This particular study discovered that 21% of the patients were diagnosed with chronotropic incompetence, a condition prevalent especially among those with persistent COVID-19 symptoms and diminished exercise capacity^5^. Aerobic exercise performance fundamentally relies on increased oxygen consumption, requiring a corresponding elevation in heart rate. Consequently, chronotropic incompetence, characterized by an inadequate heart rate response, emerges as a key factor in exercise intolerance during the initial months post-infection^8^.

Our research further elucidated the linkage between chronotropic incompetence in post-COVID-19 patients and a sedentary lifestyle, which is exacerbated by persistent symptoms such as dyspnea and fatigue. An inadequate heart rate response during exercise could lead to diminished peripheral muscle perfusion, pointing to potential autonomic dysfunction^9^. These findings underscore the importance of timely diagnosis and therapeutic intervention, as chronotropic incompetence not only predicts cardiovascular morbidity and mortality but may also underlie early exercise-related fatigue. Hence, targeted interventions, including cardiopulmonary rehabilitation programs, are vital, as they have shown efficacy in enhancing aerobic capacity and the quality of life for post-COVID patients with chronotropic incompetence^10^.

This study, while providing valuable information on chronotropic incompetence in long COVID, has several limitations. Its cross-sectional design allows for the identification of associations but not causality, limiting our ability to infer the directionality or temporal sequence of the observed findings. Conducted at a single private hospital in Sergipe, Brazil, the research might face challenges in generalizability due to the specific geographic and healthcare context. Another limitation is the reliance on self-reported physical activity levels to define sedentary behavior, which could lead to reporting bias and lacks the objectivity of direct measurement. Finally, while the findings resonate with existing literature, the unique context and characteristics of our study population need to be considered when comparing these results with other studies, as there might be significant differences in patient demographics, healthcare access, and management strategies.

In summary, approximately 20% of individuals suffering from post-acute COVID-19 syndrome in our study exhibit chronotropic incompetence, which is associated with decreased aerobic capacity and increased sedentary behavior. Recognizing and understanding this association is pivotal for the development of effective therapeutic strategies aimed at enhancing patient well-being, an endeavor that grows increasingly important as the global community continues to confront the ongoing challenges of the pandemic.
